# From the search for diagnosis to treatment uncertainties: challenges of care for rare genetic diseases in Brazil

**DOI:** 10.1590/1413-812320182410.01612019

**Published:** 2019-09-26

**Authors:** Jorge Alberto Bernstein Iriart, Marina Fisher Nucci, Tatiane Pereira Muniz, Greice Bezerra Viana, Waleska de Araújo Aureliano, Sahra Gibbon

**Affiliations:** 1 Instituto de Saúde Coletiva, Universidade Federal da Bahia. R. Basílio da Gama s/n, Canela. 40110-040 Salvador BA Brasil; 2 Casa de Oswaldo Cruz, Fiocruz. Rio de Janeiro RJ Brasil; 3 Universidade Federal do Rio Grande do Sul. Porto Alegre RS Brasil; 4 Programa de Pós-Graduação em Ciências Sociais, Universidade Federal da Bahia. Salvador BA Brasil; 5 Instituto de Ciências Sociais, Universidade do Estado do Rio de Janeiro. Rio de Janeiro RJ Brasil; 6 Anthropology Department, University College of London. London England

**Keywords:** Rare disease, Genetics, Therapeutic itineraries

## Abstract

Rare genetic diseases are an important public health problem, but they are still little studied in Collective Health. This article aims to analyze the ‘therapeutic itineraries’ of patients in search of a diagnosis and treatment for rare genetic diseases in the cities of Rio de Janeiro, Salvador and Porto Alegre. It focuses on the material challenges, emotional and structural problems faced in these trajectories. Semi-structured interviews were conducted with patients/caregivers and health professionals in the context of public health medical genetics. Our findings suggest that the experience of the rare genetic disease is aggravated by practical, inter-relational and bureaucratic/institutional problems. The reality of long and circuitous journeys to obtain a diagnosis, non-geneticists’ lack of knowledge about rare diseases, difficulties in transportation and access to specialists, diagnostic and complementary examinations, and access to high-cost medicines and food supplies were common challenges in all the narratives examined in the three Brazilian cities. In addition, adherence to care provided by medical genetics requires action and strategies that depend on arrangements involving family members, physicians, patient associations, and the state.

## Introduction

*“*[…] *Look, I’m sorry to tell you, but your*
*daughter never had autoimmune hepatitis*.
*She has another disease that I have not yet*
*discovered, but we are going to find out what it is*
[…]*” (report of the caregiver of the two sisters with*
*Type I tyrosinemia, Porto Alegre)*

Rare genetic diseases are an important public health problem, but they are still rarely studied from the perspective of Public Health in Brazil. A brief research carried out in the SciELO data base with descriptors *doença genética rara* or *doenças raras* returned 65 and 1,143 references, respectively, of which only six were part of the *Saúde Pública* collection. The Ministry of Health^1^ defines rare disease as one that affects up to 65 people out of every 100,000 individuals. Each rare disease, taken separately, affects a limited number of people. Considering, however, that around 6,000 to 8,000 different types of rare diseases are found worldwide, when grouped under one category, their epidemiological impact is quite significant. The number of patients affected by a rare disease can reach 30 million people in Europe and 25 million in North America^2^, with prevalence in the population of around 6-8%^3^. In Brazil there are an estimated between 13 and 15 million people with a rare disease.^4^ With declining mortality from other causes due to improved maternal and child health in the last decades, rare diseases have become the second cause of child mortality^5^.

Rare diseases are characterized by varying signs and symptoms not only among different diseases but also among patients with the same disease^1^. About 80% are caused by genetic factors, and the rest by environmental, infectious and immunological factors, among others^1^. They are usually chronic, progressive and incapacitating, and may also be degenerative causing physical, mental, behavioral and sensory changes. Treatment often requires multidisciplinary follow-up by medical geneticists, physiotherapists, speech therapists, nutritionists and psychologists, among others, to alleviate symptoms or delay their onset. Most rare diseases have no effective treatment. It is estimated that only 10% of them have some specific drug treatment that often incurs a very high cost^4^.

Rare genetic diseases can have a significant impact on the quality of life of those affected and their families who are often faced with a lack of information about the disease, stigma and prejudice, and also demands for care that often leads to one of the parents to finish paid work to provide care exclusively for their sick child^6^. Among the various problems that afflict patients and their families in coping with rare genetic diseases is also the diagnosis which is often made more difficult by many signs and symptoms being similar to those of common diseases.

Brazilian genetic medical practice is recent, and the first medical residency was created at the Hospital das Clínicas of Ribeirão Preto (USP) in 1977^7^. Despite several initiatives in recent decades to include care for rare genetic diseases in the public health system (SUS), this integration is still insufficient. Many of the medical genetic services belong to public universities and reference hospitals concentrated in the South and Southeast regions of Brazil.^7^ The number of medical geneticists in the country is low and does not exceed 250 professionals, which is insufficient for universal access to this speciality^8^. Brazilian studies on genetic care point to problems such as the centralization of services in large urban centers, problematic access to specialized services and the almost non-existence of geneticist physicians in SUS, working outside reference centers or university hospitals^5^.

In 2014, the National Policy for the Comprehensive Care of People with Rare Diseases (PNAIPDR)^9^ was implemented aiming to incorporate rare genetic diseases into the SUS, with the goal of integrating genetic services into primary care and with regional reference centers. The PNAIPDR provided, in theory, the basis for the establishment of reference centers that would receive funding for genetic testing, an expanded list of drug supply and the provision of genetic counseling^4^.

Against this background context, this paper aims to analyze the therapeutic itineraries of patients and their families in search of diagnosis and treatment for rare genetic diseases in three capitals in different Brazilian regions, focusing on the paths they travel through the biomedical field and the material, emotional and structural challenges of these journeys. This is a subject rarely studied in Brazil. There are practically no studies about the therapeutic itineraries of patients with rare genetic diseases in the country, moreover the recent enactment of the PNAIPDR enables close observation how this policy is affecting (or not) the daily life of patients and caregivers. Given the relevance of the theme to Public Health, it is vital to know the health needs of this particular group of patients and their caregivers, their perceptions of the therapeutic process, and the hardships and obstacles they face in accessing diagnosis and treatment.

For the analysis undertaken in this paper, we consider that the notion of ‘therapeutic itineraries’ refers to the paths taken by patients and caregivers to deal with distress, their experience of the treatments, the meanings assigned to illness and the availability and accessibility of care resources^10,11^. The search for treatment is a complex elective process influenced by the sociocultural context^12^ in which it is important to consider the interpretation of the subjects’ experiences and their actions regarding macro-social processes that inform this pathway to care^11^. Therefore, the analysis is not limited to just this actual pathway but also includes the experience of patients and caregivers in day to day living with disease, which in the case of rare genetic diseases implies continuous care.

## Methods

A qualitative, multicenter, anthropologically-oriented study was conducted with semi-structured interviews with health professionals and patients and caregivers of patients with rare genetic diseases in the cities of Rio de Janeiro, Salvador and Porto Alegre. Cities were selected in different regions of the country to analyze the differences, similarities, barriers and facilities in the therapeutic itineraries of patients or caregivers with rare genetic diseases. Of the three cities, Salvador has the lowest number of geneticists, only 9, compared to 24 in Rio de Janeiro^13^. On the other hand, Porto Alegre has 34 medical geneticists^13^ and is considered a reference center for genetics in Brazil. It is common that requests for genetic testing from other states are sent there to be performed.

Fieldwork took place from August 2017 to February 2018 and was funded by the Wellcome Trust, UK. The project was approved by CONEP and by the ethics committees of all institutions that participated in the research. All recommendations of Resolutions CNS 466/12 and CNS 510/16 were followed.

### Research participants

The research was conducted at genetics clinics of public hospitals (two in Rio de Janeiro, one in Salvador and one in Porto Alegre) and a non-profit health care institution (in Salvador).

Twenty-eight semi-structured interviews were conducted with patients and caregivers (9 in Salvador, 10 in Porto Alegre and 9 in Rio de Janeiro). Rare diseases are very varied in their clinical manifestation, and we tried to select patients from several outpatient clinics and with different diagnoses. The inclusion criteria were: 1. Being under care in the public service of medical genetics or being a caregiver of a patient receiving care; 2. Having full cognitive ability and 3. Being available and willing to agree to participate in the research. The selected patients had several diagnoses, namely, Marfan syndrome, Ehlers-Danlos syndrome, sexual developmental anomaly, pigment incontinence, hereditary metabolic disorders due to inborn metabolism errors, such as maple syrup urine disease, tyrosinemia, mucopolysaccharidosis, Gaucher’s disease, Niemann-Pick’s disease, phenylketonuria and cystic fibrosis. Some patients still had no conclusive diagnosis (Chart 1).

Of the 28 patients interviewed, seven had a private health plan (three in Rio de Janeiro, three in Porto Alegre and only one in Salvador) and 15 did not reside in the capitals and had to move from their municipalities to receive care. A patient moved from his home state to Porto Alegre to perform the treatment.

We also conducted 28 semi-structured interviews with health professionals (10 in Salvador, 10 in Porto Alegre and 8 in Rio de Janeiro) who work in medical genetics services, addressing their views on the difficulties faced by patients in accessing and treating rare genetic diseases. The interview respondents were mostly Medical Geneticists (n = 19). Endocrinologists, Neurologists, Oncogeneticists, Nurses, Biologists, Nutritionists and Psychologists have also been interviewed (Chart 2).

The interviews were conducted in rooms available at the hospitals participating in the research in the three cities. In Rio de Janeiro and Porto Alegre, due to the lack of rooms available, some interviews were held in the waiting room of genetics outpatient clinics. In Porto Alegre, three interviews were held during medical visits. All the interviews were digitally recorded and fully transcribed.

### Data analysis

Thematic content analysis was performed using the NVivo 11 software to assist in the codification and management of the database. A tree of analytical categories was designed to guide data coding. The analysis sought to identify relevant themes in the interviews and to analyze and interpret the therapeutic itineraries of the patients in the light of their sociocultural contexts^12,14^. Given the similarities in the results found in the three research contexts, we decided to present them together throughout the text.

## Results

### The long and winding journey to diagnosis

The journey of several patients to the genetic services of the centers of reference investigated is very varied, due to the large number of genetic diseases and their different signs and symptoms. In some cases, the pathway was short with a suspected genetic problem and rapid referral by primary care pediatrician or by a medical specialist to the genetic service. However, in all three research contexts, the situation of many patients was significantly marked by what might be described as a long ‘pilgrimage’ to several specialists in search of a diagnosis.

Health professionals and caregivers interviewed confirm that this can be attributed mainly to the lack of knowledge of PHC professionals about rare genetic diseases. The foreign nature of such diseases contributes to the lack of knowledge by professionals, who have heard little or nothing about the subject in their educational training and therefore do not suspect a genetic disease. Referral to the genetics service was referred to as more difficult by patients residing in the interior of the state. Geneticists state that general practitioners or pediatricians in primary care of inland cities are unaware of the existence of the genetics service in the capitals.

Referring to mucopolysaccharidosis, a geneticist from Salvador reports:

The patient will see several specialists to achieve a diagnosis. We did studies here of diagnostic age. I can’t tell you precisely the age of our patients in the diagnosis, but surely a good part was over ten years old. So most went through those ten years there, with symptoms, visiting pediatricians, ophthalmologists, cardiologists, otolaryngologists without a diagnosis. (geneticist 04, Salvador)

A typical report of patients is a history of going back-and-forth between different health facilities medical specialties and of wrong or late diagnosis that can result in the rapid advancement of diseases due to lack of treatment or the use of inadequate medicines, This can further aggravate the general state of health and leave irreparable sequelae. An emblematic case, reported in Porto Alegre, was that of parents of two children with Type I Tyrosinemia who only discovered that they had a rare disease from the diagnosis of liver cancer of the younger sister aged one year and three months.

*The doctor diagnosed her as if she had autoimmune hepatitis, so she came to treat autoimmune hepatitis* … *right after she was born, she was just over a year old or so. Then* … *she was growing up and developed rickets, developed other diseases. However, we did not know why* … *what was causing these other diseases*. […] *But then she was referred to São Paulo* … *I contacted a hepatologist from São Paulo, and she told me that my daughter never had autoimmune hepatitis, but another disease that she hasn’t discovered yet*. (Caregiver 09, Porto Alegre)

Due to misdiagnosis, the oldest daughter has a set of sequelae associated with the development of type I tyrosinemia, including rickets, at the age of five.

In another case reported by a medical geneticist in Salvador, the child was born very small, the mother noticed a delay in development and sought the pediatrician. The doctor told her that “every child grows in their own time” and that there was no abnormality. She then sought a neurologist who requested several tests, and she was only referred to the genetics service when the child was two years and seven months old. The geneticist said this was a case of metabolic disease that could have been diagnosed only with anamnesis, avoiding a series of unnecessary tests.

The late diagnosis meant losing a window of opportunity for early intervention that could have provided a good clinical evolution because when the patient arrives at the genetic service, the disease is already at a stage where treatment is no longer indicated or is ineffective. Errors and late diagnosis of rare genetic diseases are frequent among non-specialists, which indicates the need for further guidance and continued training of the medical staff^15^. The teaching of genetics in Brazilian medical courses is usually limited to a single discipline of medical genetics, and is optional in some universities^13^, meaning that physicians can complete their course of training without a minimum knowledge in genetics.

According to the geneticists interviewed, the deficiencies in the care network and flow of care are factors that also contribute to a delayed diagnosis, since doctors initially must request tests that allow them to exclude the most common diseases. Patients’ difficulty in accessing straight-forward exams, such as an echocardiogram, also contributes to late diagnosis and referral to a geneticist.

Getting to a reference centre in genetics means for some patients the end of an anguished journey in search of a diagnosis and consequent onset of treatment. This is permeated by various expectations and anxieties, as reported by patients in the three cities surveyed. Patients say that exposure to genetic idiom occurs for the first time upon arrival in the service and many do not even know what to expect because it is their first contact with information about a genetic disease. Many of them expect a conclusive diagnosis and the onset of treatment, while others hope that the diagnosis for a rare genetic disease is not confirmed.

The need for diagnosis in cases of genetic diseases combines two current values in health care; on the one hand, the predictive character of genetics and, on the other hand, risk management for both the individual and subsequent generations. The concept of health risk becomes a significant predictor for a set of interventions, whether about specific social groups or individuals, so that determining risk factors becomes one of the main tasks of contemporary medicine^16^.

For professionals and patients, the hereditary nature of rare genetic diseases points to the need for information and accountability for the proper orientation of future reproductive decisions. Genetic counseling is a central concern for medical genetics professionals, which has reflected on the training and need requirements of regulation of the profession in Brazil^13,17^.

Kinship and heredity are common themes in patients’ reports, given that some obtained their diagnosis through the diagnosis of their siblings, even when there are still no symptoms that lead to a suspected health problem. Many of the rare genetic diseases are autosomal recessive, that is, they depend on the genetic contribution of the father and the mother, and are potentiated in cases of consanguinity. Kinship is also evidenced in patients’ reports about the way professionals approach the subject.

The arrival at the genetics service is essential for a family’s orientation on reproductive options, especially in the case of autosomal recessive diseases such as Duchene’s and most hereditary metabolic diseases, which depend on the inheritance of identical genes of the father and the mother, and these do not have the disease, but children are likely to manifest them. However, the guidelines for the monitoring of rare genetic diseases in the SUS do not currently include the monitoring of family members, besides informing them about the risks. Many genetic diseases have no available treatment or medication, but being accompanied by a multidisciplinary team provides caregivers and patients with emotional support and accompanying guidance that are critical. Despite all the difficulties related to infrastructure, genetics services are consistently been praised by patients and caregivers, especially regarding the reception and proper care of the professionals in the three research sites.

### Difficulty in accessing specialists and diagnostic and complementary examinations

For patients the journey to diagnosis is often made up of private health care visits and examinations until the patients can access the genetics service in the SUS; it is this point which triggers a series of extra costs for a population that is generally low-income. In the three cities surveyed, patients usually used private health care providers to access specialists and diagnostic exams which are difficult or time-consuming to access in SUS. This difficulty in accessing complementary examinations and diagnostic tests continues well after patients’ arrivals at a genetics service. Some people resort to raffles and family-friends fundraising initiatives to pay privately for the examinations and doctors.

In the face of the degenerative and crippling nature of many rare genetic diseases, many patients normally should undergo several early therapeutic interventions because of the need for motor, sensory, and cognitive stimuli. Although SUS guidelines for the comprehensive care of patients with rare diseases outline the need for multidisciplinary care of these patients, access to the various professionals is still fragile, due both to the scarce material and human resources and the obstacles set by the regulatory system. Just because a patient is followed-up by a geneticist this does not give him treatment priority by other specialists, and patients must return to the system queue for each specialty. In Rio de Janeiro, for example, the functioning of SISREG, a state regulation system, is cited by most of the medical team as a hindrance to patient care:

*Because SISREG does not look at the hospital like a hospital. It’s like we’re a health post or something*. […] *Cardiology is right at the corner, but I cannot send the patient there*. […] *What happens is that the patient theoretically goes to the referral system and never returns. Because they’re going to have him do the blood count test in the West Zone, then they’ll send him to Mauá Square*…*and the patient never returns*. (Geneticist 03, Rio de Janeiro)

Likewise, the system’s limits do not allow for extending care to families who, in the particular case of genetic diseases, have a high probability of having other similar cases that would need follow-up. Professionals emphasize that, besides compromising their autonomy, bureaucratic limits prevent potential service use.

The centralization of reference hospitals in the capitals and the concentration of highly complex treatments in the south and southeast regions of the country sometimes leads to the need to move the whole family to other states, often leading to unemployment and reorganizing life in another city.

Primary care unit professionals’ lack of knowledge about rare genetic diseases is pointed out by patients and caregivers as yet another difficulty they face when seeking clinical care, after diagnosis, in their municipalities of residence. As evidenced by the statements of the interlocutors, professionals feel insecure in prescribing some medication that can aggravate the picture of patients with genetic diseases, which hinders treatment at the local level, forcing patients to move to the capitals.

The difficulty of transportation, that often have to be demanded from the municipalities, to commute to the genetics services or other specialties were frequently cited in the interviews as one of the main issues faced by patients in the three study sites. Several caregivers interviewed face many travelling hours to come to the visits. The longest route was reported in Salvador by a caretaker who traveled eight hours to get to the visits. Patients coming from the interior of the state usually leave their hometown at dawn (some report leaving at 2 a.m.) and arrive in the morning at the hospitals of the capital. However, they are often seen only in the afternoon shift and have to fund food and travel around the city for specific tests, such as a child electrocardiogram, which sometimes has to be performed in another hospital. Caregivers and doctors report the pressure from transport drivers made available by the municipality who often insist that caregivers and patients to return to their cities in the early afternoon.

“You have to leave; you have to leave! Or else, I’ll drop you here!” So they leave some people behind. I’ve seen a patient crying; I had to borrow a cell phone, try to call the inn, to know what to do, you know? (Geneticist 02, Salvador)

### Care: lack of caregiver support and overload

Caregivers have a fundamental role to play in the therapeutic itineraries of patients with rare diseases because, in general, these diseases make people less autonomous and more dependent, requiring the constant presence of the caregiver who takes patients to health services, evaluates therapeutic options, provides cares for them on a daily basis; this often entails changing their lives to attend people they care for. This care usually falls on women, especially mothers, who are often seen as “naturally” destined and responsible for this task^18^. Thus, if childcare itself is already viewed socially as the “natural” and “instinctive” responsibility of the mother, in cases where specific care is needed, this pressure becomes even more pronounced^19^. The caregivers interviewed reported that the lack of domestic support is a problem since the demands and tasks that the care of these patients require are strenuous in their daily life.

The inquiry about care includes epistemological, political and moral presuppositions for the production of well-being, health and citizenship in our societies^20,21^. Thus, its analysis allows us to look at the therapeutic path of different diseases.

Several studies in social sciences systematize how care has been devalued in Western societies because it is associated with emotions, intimacy and subordinate social sectors: women, the poor, ethnic minorities, etc.^20,21^. Care is therefore at the same time moral, relational and historically specific^22^, and should be understood as a complicated process that consumes energy, time and financial resources, in which knowledge, technologies, tasks and bodies intervene^21^.

The question about care involves epistemological, political and moral assumptions involved in the production of welfare, health and citizenship in our societies^21^. Its analysis allows us, therefore, another look at the therapeutic trajectory of different diseases.

In the scenario of rare genetic diseases, this care is not only directed to a specific individual but the family as a whole in their projects of existence and reproduction^4^. In the face of the diagnosis of a rare hereditary disease, several people will be included in the investigation, such as siblings, cousins, nephews, for example. Also, the duration and consequences of a rare disease usually require extensive care involving other family members, especially mothers. Unlike the diagnosis of other diseases, the diagnosis of rare hereditary disease traverses several bodies, *either through the genetic component that has been (or can be) transmitted or inherited, and which cannot be controlled or altered by the actions of the subject, or the debilitating physical conditions that will require the care of others until the end of the patient’s life*^4^.

In the case of caregivers and patients with genetic diseases who are required to follow restricted diets, the most significant challenges are related to daily necessities, such as compliance with a limited diet and the unavailability or high cost of adequate food products, as well the management of care marked by the anguish of often not knowing what is allowed and forbidden in the children’s diet, the emotional distress of having to deny them particular wishes and administer what is or is not allowed to share with the brothers who do not have the disease, besides problems related to food outside the home, at school or at birthdays and family celebrations.

Financial aid or income supplementation policies were highlighted in the interviews as a necessity, given that caregivers must frequently give up their work to care for patients. Unemployment and financial difficulties were frequently cited:

*Our difficulty is that we are unemployed. So she* … *We’re overriding everything to do all his exams, to come here*[…]. *For example, asking others for help, because we are going through some financial constraints. We can only solve everything here*. (Caretaker 02, Rio de Janeiro)

### Difficulties in accessing high-cost medicines and food supplies

Many of the rare genetic diseases are associated with the deficient production of certain central metabolism enzymes, so that the treatment also consists of enzymatic replacement, besides the regular exams and clinical follow-up. In the case of rare diseases, the drugs required for treatment are incredibly high-cost, since they are associated with the pharmaceutical industry’s production of orphan drugs.

The difficulties of access these drugs is because the premise of rarity is associated with the low-profit potential of industries that would not be interested in developing drugs for a minimal number of consumers. Thus, according to Schwartz et al.^7^, two concepts are taken together to assign a drug status to an orphan drug, namely, the epidemiological (prevalence or incidence of a disease within a population) and the economic (the presumed non-profitability of the drug for the treatment of the disease)^7^.

Given the complex process for registering and incorporating high-cost medicines into SUS, involving industry and state interests, many patients find judicialization the only way to access drugs or genetic tests^23^. Of the 28 patients/caregivers interviewed, 12 went to court to obtain medication or tests.

*To date she has not received the medication. Because the population with mucopolysaccharidosis has an increase in the liver and spleen. You need medication to control it. So to this day, she could not get it*. […] *She already filed a couple of requests [at the Public Prosecutor’s Office], but they do not issue an order, they do not authorize it*. (Caregiver 09, Salvador)

Legal processes to obtain medicines indicate a deficiency in the rare disease care policy, with consequences for the health system, such as higher expenses^24^. This pathway is not seen as the best route either by patients, who are always jolted by the uncertainty if they will receive the medication, or by doctors, who point out that judicialization stigmatizes patients with rare diseases as a problem-patient for the State.

*So we have a lot of sad cases like this. The patient judicializes, wins, then begins to use the medication. Then the state interrupts for some reason, because it is out of funds, because it did not bid, because the bidding was delayed, and so the patient dies because of a [lack of] medicine, a diet* … *it is very exhausting, it is very harmful*. (Geneticist 05, Salvador)

Alongside the emotional cost of judicialization is the cost of bureaucratic demands placed on health professionals monitoring patients with rare diseases. From evidence and efficacy concepts, the Judiciary repeatedly requires medical reports that attest patients’ demands. However, the understanding of health and rights does not always coincide.

In the case of inborn errors of metabolism, for example, this difficulty lies in the distinction between food and medicine. Treatment with starch or food supplement formulas is essential for the treatment of some diseases. They function as medications that underpin the treatment of replacement of nutritional substances that individuals with some metabolic disease do not produce or do so insufficiently. The understanding of the Judiciary in these cases is that these items are foods and not drugs and, therefore, cannot be provided by legal means.

Some patients/caregivers, just because their conditions are rare, believe it would be easier for the government to pay more attention to then and facilitate treatment more quickly. However, from the institutional viewpoint of the SUS, and the Judiciary, which acts more directly in the ruling regarding the right of access to health care, meeting the specificity of rare diseases confronts the debate about the universality principle of the SUS. This principle is often interpreted as broad coverage to the detriment of the notion of equity in meeting health specificities – besides the ethical debate on the justice of the decision to direct resources to a high-cost treatment for the few, as opposed to apply resources in the treatment of diseases that affect more people.

In the case of metabolic diseases, concern about diet is fundamental to treatment, so patients often do not have the financial resources to follow what is recommended. Thus, resorting to treatment through judicialization is often necessary, as well as other alternatives such as help from family members, organization of campaigns through social networks, association and creation of support groups at the national level (given the small number of cases).

## Conclusion

Our results show that the difficulties and anxieties of caregivers and patients vis-à-vis rare genetic diseases confront in their therapeutic itineraries (despite their variety and non-linearity) are similar in the three cities studied. Long-term therapeutic pathways to diagnosis, non-geneticists’ lack of knowledge about rare diseases, difficulties in transportation and access to specialists, diagnostic and complementary exams, and the access to high-cost medicines and food supplies were common to narratives. Caregivers, mostly women, are overwhelmed, and develop various strategies, not infrequently having to resort to the private sector to enable access to care for patients. Adherence to care provided, often summarized as symptom control rather than cure, requires strategies that rely on arrangements involving family members, physicians, patient associations, and the state.

The public health system in Brazil has faced in recent years an increasing process of underfunding and precariousness^25^. The recent fiscal austerity policy has also resulted in cuts to the budget of social policies and programs aimed at the most vulnerable population^25^. In this sense, some of the difficulties faced by caregivers and patients with rare genetic diseases are shared by other SUS patients. There are, however, specific difficulties in accessing comprehensive care for patients with rare genetic disease in SUS, as evidenced in our results, which precede the political and economic crisis and could be alleviated with the effective implementation of the PNAIPDR enacted in 2014. At the moment, only a few referral centers have been accredited and many have not yet received the resources they expected. Our results show that PNAIPDR has not yet had significant repercussions in the lives of patients and caregivers.

The qualification and training of health professionals in primary and specialized care for the diagnosis and treatment of rare genetic diseases in order to prevent late diagnosis and provide follow up treatment in primary care (preventing the need for patients to travel to the state capitals), are fundamental actions that are provided for in the policy, as well as access to genetic testing and medicines. It is also crucial that medical genetics be a compulsory discipline in all medical courses, aiming to provide physicians with basic training in genetics. The effective implementation of PNAIPDR is essential for patients with rare genetic diseases to have greater access to comprehensive care in the public health system.

## Figures and Tables

**Chart 1:** Patients / Caregivers data.

	Patient or caregiver	City of interview	Municipality of residence	Hospital / Institution	Diagnosis	Sexual Differentiation Anomaly (SDA) is a situation in which sex (46, XX or 46, XY), gonadal sex (ovary or testis) and phenotype sex (male or female appearance) are not in agreement.	Private Health Plan	Judicialization
E1	Patient	Salvador	Salvador-Ba	Hospital 1	Sexual ambiguity	-	No	No
E2	Patient	Salvador	Salvador-Ba	Hospital 1	Doesn't know the diagnosis	A rare genetic disease (0.7/100,000 births) of dominant X-linked inheritance, in which 97% of the affected are women and is almost always fatal in males, leading to spontaneous abortion in most cases. The main symptoms are skin lesions in varying degrees.	No	No
E3	Caregiver	Salvador	Santo Estevão-Ba	Hospital 1	Pigment Incontinence	-	No	No
E4	Caregiver	Salvador	Piatã-Ba	Hospital 1	Suspected neuromuscular disease	It is a connective tissue disease characterized by joint hypermobility, hyperextensible skin and abnormal healing. The skin is flaccid and elastic, showing marked hyperextensibility. The wound scars are enlarged, papyraceous, especially on the knees and elbows.	No	No
E5	Caregiver	Salvador	Santa Inês-Ba	Hospital 1	Ehlers-Danlos Syndrome	MPS is part of the inborn errors of metabolism (IEM) group. They are caused by the deficiency of specific lysosomal enzymes, which affect the catabolism of glycosaminoglycans. MPS standard features include progressive thickening of features, corneal opacification, recurrent airway infection, liver and spleen enlargement, valvular heart disease, joint stiffness/abnormalities, and growth alterations, among others.	No	No
E6	Caregiver	Salvador	Salvador-Ba	Hospital 1	MPS6	It is an autosomal dominant disease that affects the connective tissue, responsible for strengthening body structures. It usually affects the heart, eyes, blood vessels and skeleton.	No	Yes
E7	Caregiver	Salvador	Salvador-Ba	Hospital 1	Marfan Syndrome (Suspected)	-	Yes	No
E8	Caregiver	Salvador	Salvador-Ba	Hospital 1	Diagnosis not available	Idem MPS	No	No
E9	Caregiver	Salvador	Lauro de Freitas-Ba	Hospital 1	MPS1	Idem MPS	No	Yes
E10	Caregiver	Rio de Janeiro	Rio de Janeiro-Rj	Hospital 2	MPS	Idem MPS	No	No
E11	Caregiver	Rio de Janeiro	Rio de Janeiro-Rj	Hospital 2	MPS	-	Yes	Yes
E12	Caregiver	Rio de Janeiro	Nova Iguaçu-Rj	Hospital 2	Diagnosis not available	It is a genetic disease that causes abnormal skeletal growth, leaving the individual 20% below the normal range of other individuals his/her age or below the average of the general population.	No	No
E13	Caregiver	Rio de Janeiro	Rio de Janeiro-Rj	Hospital 2	Stunting (no specific diagnosis)	-	No	No
E14	Caregiver	Rio de Janeiro	Miraí-Rj	Hospital 2	Diagnosis not available	CF is a multisystemic genetic disease with an autosomal recessive inheritance that affects the epithelium of the respiratory tract, pancreas, intestine, hepatic biliary system, sweat glands and male deferent ducts.	No	Yes
E15	Caregiver	Rio de Janeiro	Rio de Janeiro-Rj	Hospital 2	Cystic fibrosis	Idem CF	No	Yes
E16	Caregiver	Rio de Janeiro	Campos dos Goytacazes-Rj	Hospital 2	Cystic fibrosis	Idem CF	Yes	No
E17	Patient	Rio de Janeiro	Rio de Janeiro-Rj	Hospital 2	Cystic fibrosis	Idem CF	Yes	No
E18	Caregiver	Rio de Janeiro	Belford Roxo-Rj	Hospital 2	Cystic fibrosis	Idem MPS	No	No
E19	Patient	Porto Alegre	Gravataí - RS	Hospital 3	MPS1	It is a disease also called hepatorenal tyrosinemia caused by the deficiency of the enzyme Fumarylacetoacetate hydrolase. It is a hereditary metabolic disease of an autosomal recessive inheritance pattern.	No	Yes
E20	Caregiver	Porto Alegre	Jaraguá do Sul - SC	Hospital 3	Tyrosinemia	Idem MPS	No	Yes
E21	Caregiver	Porto Alegre	São Leopoldo - RS	Hospital 3	MPS4	Idem MPS	No	Yes
E22	Caregiver	Porto Alegre	Santo Antônio da Patrulha - RS	Hospital 3	MPS4	Gaucher disease (GD) is the most frequent lysosomal disease. Hepatosplenomegaly, anemia, thrombocytopenia, bone pain, osteopenia and growth retardation are the most common clinical manifestations	No	Yes
E23	Patient	Porto Alegre	Porto Alegre	Hospital 3	Gaucher disease	Idem GD	Yes	Yes
E24	Patient	Porto Alegre	Porto Alegre	Hospital 3	Gaucher disease	Idem DG	Yes	No
E25	Caregiver	Porto Alegre	Porto Alegre (veio de Pernambuco)	Hospital 3	Maple syrup urine disease (MSUD)	The Maple Syrup Urine Disease (MSUD), also known as Leucinosis, is an Inborn Error of Metabolism caused by the deficiency of thiamine-dependent branched-chain α-ketoacid dehydrogenase complex (BCKDC) activity. The accumulation of these amino acids mainly affects the central nervous system (CNS).	No	Yes
E26	Caregiver	Porto Alegre	Boa Vista do Buricá - RS	Hospital 3	Phenylketonuria	Idem CF	No	No
E27	Caregivers	Porto Alegre	Canoas - RS	Hospital 3	Tyrosinemia type 1	Idem Tyrosinemia	No	Yes
E28	Caregiver	Porto Alegre	São Leopoldo - RS	Hospital 3	Niemann-Pick disease (Type B)	The Niemann-Pick disease consists of a group of sporadic genetic syndromes that are inherited within the same family and that cause lipid accumulation in some organs such as the brain, spleen or liver, for example. Type B is a less severe form of type A that allows survival to adulthood.	Yes	No

Source: Own elaboration. Data on rare diseases^15^.

**Chart 2:** Health professionals.

Professional	City	Professional qualification/employment contract	Specialty
P1	Salvador	Doctor/Professor	Geneticist
P2	Salvador	Biologist/Researcher	-
P3	Salvador	Doctor	Geneticist
P4	Salvador	Doctor	Geneticist
P5	Salvador	Doctor/Professor	Endocrinologist
P6	Salvador	Doctor	Geneticist
P7	Salvador	Doctor	Neurologist
P8	Salvador	Psychologist	-
P9	Salvador	Doctor	Geneticist
P10	Salvador	Doctor	Geneticist
P1	Rio de Janeiro	Doctor	Geneticist
P2	Rio de Janeiro	Doctor	Geneticist
P3	Rio de Janeiro	Nurse	-
P4	Rio de Janeiro	Psychologist	-
P5	Rio de Janeiro	Doctor	Geneticist
P6	Rio de Janeiro	Doctor	Neurologist
P7	Rio de Janeiro	Doctor	Geneticist
P8	Rio de Janeiro	Doctor	Geneticist
P1	Porto Alegre	Doctor	Geneticist and Biochemist
P2	Porto Alegre	Doctor	Geneticist
P3	Porto Alegre	Doctor	Geneticist and Clinical Pathology
P4	Porto Alegre	Doctor Resident	Geneticist and Pediatrician
P5	Porto Alegre	Doctor	Geneticist/Neurogeneticist
P6	Porto Alegre	Doctor/Professor	Geneticist
P7	Porto Alegre	Doctor Resident	Geneticist
P8	Porto Alegre	Nutritionist	Phenylketonuria
P9	Porto Alegre	Doctor/Professor	Geneticist
P10	Porto Alegre	Nurse	Childcare
